# Community referral in home management of malaria in western Uganda: A case series study

**DOI:** 10.1186/1472-698X-6-2

**Published:** 2006-03-16

**Authors:** Karin Källander, Göran Tomson, Jesca Nsungwa-Sabiiti, Yahaya Senyonjo, George Pariyo, Stefan Peterson

**Affiliations:** 1 Division of International Health (IHCAR), Department of Public Health Sciences, Karolinska Institutet, 17177 Stockholm, Sweden; 2 Medical Management Centre (MMC), Karolinska Institutet, 17177 Stockholm, Sweden; 3 Makerere University Institute of Public Health, Kampala, Uganda; 4 Department of Pharmacology and Therapeutics, Makerere University, Kampala; 5 Uganda Ministry of Health, Division of Child Health, Kampala, Uganda; 6 Department of Paediatrics, Mulago Hospital, Kampala

## Abstract

**Background:**

Home Based Management of fever (HBM) was introduced as a national policy in Uganda to increase access to prompt presumptive treatment of malaria. Pre-packed Chloroquine/Fansidar combination is distributed free of charge to febrile children <5 years. Persisting fever or danger signs are referred to the health centre. We assessed overall referral rate, causes of referral, referral completion and reasons for non-completion under the HBM strategy.

**Methods:**

A case-series study was performed during 20 weeks in a West-Ugandan sub-county with an under-five population of 3,600. Community drug distributors (DDs) were visited fortnightly and recording forms collected. Referred children were located and primary caretaker interviewed in the household. Referral health facility records were studied for those stating having completed referral.

**Results:**

Overall referral rate was 8% (117/1454). Fever was the main reason for mothers to seek DD care and for DDs to refer. Twenty-six of the 28 (93%) "urgent referrals" accessed referral care but 8 (31%) delayed >24 hours. Waiting for antimalarial drugs to finish caused most delays. Of 32 possible pneumonias only 16 (50%) were urgently referred; most delayed ≥ 2 days before accessing referral care.

**Conclusion:**

The HBM has high referral compliance and extends primary health care to the communities by maintaining linkages with formal health services. Referral non-completion was not a major issue but failure to recognise pneumonia symptoms and delays in referral care access for respiratory illnesses may pose hazards for children with acute respiratory infections. Extending HBM to also include pneumonia may increase prompt and effective care of the sick child in sub-Saharan Africa.

## Background

More than 4.4 million children die every year in Sub-Saharan Africa where malaria and pneumonia are leading causes of death [1]. The decline in mortality rates here is slower than in other continents and the deceleration is worse among the poor [2]. Most children die at home without prior contact with the formal health sector [3]. Constrained health systems and non-functional referral strategies are major obstacles for effective primary health care delivery; both essential to curb the under-five mortality and achieving Millennium Development Goals [4].

To increase prompt presumptive treatment of malaria Uganda introduced Home Based Management of fever (HBM) [5]. Two volunteer drug distributors (DDs) per village are trained for three days and supervised by the local health centre In-charge in fever management in under-five children. Pre-packed antimalarials (currently Chloroquine + Sulphadoxine/Pyrimethamin as per national policy) are distributed free of charge in age-specific doses called Homapak. DDs record children by name, age, location, name of caretaker, date, time of fever onset and whether the child fulfils any referral criteria (convulsions, loss of consciousness, severe vomiting, not able to drink or breast feed, unable to sit or stand, difficulty in breathing, a child who has not improved after two days or who has deteriorated after Homapak treatment, and children less than 2 months or above 5 years of age). If a child presents with any of these signs the DD should immediately give verbal referral advice to seek care from the nearest health centre – in line with intervention model 3 in a recent review of CHW management models [6]. For cough or diarrhoea, the DD should recommend seeking formal care. After 3 days the DD should follow-up the child in the household and if no recovery is observed, advise on referral. For children who had been referred, the DDs should record the outcome. Referral slips are not used and "counter-referral" with feedback from health facilities to DDs is not practiced in the current format of the strategy. The strategy was designed to benefit the poor and has proven effective in reducing child mortality [7], morbidity [8] and has gained acceptance among rural mothers [9] Similar strategies are now spreading across sub-Saharan Africa [11].

Although it is believed that HBM will contribute to reduced delays in treatment and improved dosage and duration of treatment [12], several community and health systems pitfalls have been identified as potential barriers for success of HBM. Follow-up data on HBM impact on community effectiveness and coverage is currently being processed and analysed (J Nsungwa-Sabiiti, personal communication), but a base-line study demonstrates that HBM does not address the complexity of local illness concepts, indicating that prompt antimalarial treatment may still not apply for all fevers [13]. Another concern is that HBM, with its heavy focus on malaria – may enforce mothers' common misconception that children with pneumonia symptoms should be treated with antimalarials [14,15]. To avoid keeping children with potentially severe disease on antimalarials in the community, a functioning referral system from village to health centre is essential. However, paediatric referral from health centres to hospital has shown to be problematic, with only 1/3 of referrals reaching a hospital in Uganda [16] and almost half of the children referred delaying more than 2 days before accessing referral care in Tanzania [17]. If HBM referral from village to health centre is similarly constrained, HBM could potentially involve risks for children with severe illness or non-malaria related conditions. We set out to determine overall referral rate, referral causes, completion and timeliness of referral and reasons for non-compliance with referral advice in HBM implementation.

## Methods

### Study population

A case-series study was conducted between June and October in 2004 in all four parishes in one sub-county of Kasese District, western Uganda, where the under-five population was approximately 3,600. The four parishes contained 20 villages which each had two community drug distributors (DDs). Kasese district is located at the foot of the Rwenzori Mountains on the boarder of Uganda and the Democratic Republic of Congo. Six health centres and one mission hospital serves the area but access is typically poor (i.e. half of the population within 5 km of a health centre), especially for people living in the mountains. Malaria is hyper-endemic and under-5 mortality rate estimated at 170/1000 [18].

### Study procedure

In the late dry and early rainy season all 40 DDs in the sub-county and all children referred from these were included and followed for a total of 528 DD weeks of observation. Three local data collectors, recruited based on literacy skills, availability during study period and familiarity with neighbourhood, visited all DDs fortnightly for collection of completed HBM recording forms with patient and referral information. Referred children were identified with help from the DD and visited in the household by the data collector after 4–14 days. The primary caretaker of the referred child was interviewed using a semi-structured questionnaire (available from first author) with questions on symptoms prompting contact with the DD, DD actions, referral timing and completion. Coping behaviour for those not following referral advice and reasons for non-compliance were explored. Out-of-pocket costs, i.e. costs for transport, consultation, admission and drugs, were investigated through caretaker reporting of expenditure invested in the referral episode. After 6 weeks two new local terms for 'fast breathing' and 'difficult breathing' were identified and added to the questionnaire. Referred children whose caretakers stated having completed referral were traced in the outpatient registry of the health facility mentioned by the caretaker to confirm attendance, timeliness in attendance, diagnosis and treatment.

### Sample size and data analysis

A total number of 96 referred children needed to be followed-up for determination of referral completion rate with an absolute precision of ± 10%. Data was entered in EpiData () and analysed in STATA8 for overall referral rate, cause of referral, referral completion rate, timeliness in referral and reasons for referral non-compliance. Frequencies and proportions were measured in percentages and cross tabulations were analysed using χ^2^. During the study there was an outbreak of chickenpox causing many referrals. Such and other non-febrile episodes were excluded from the analysis. Repeated referrals in the same child did not occur in the study period but would have been excluded had they occurred.

### Ethical approval

The study was approved by Makerere University and Karolinska Institutet Ethics Committees (Dnr 03–693), district authorities and village chiefs. Caretakers gave informed consent.

## Results

**Figure 1:**
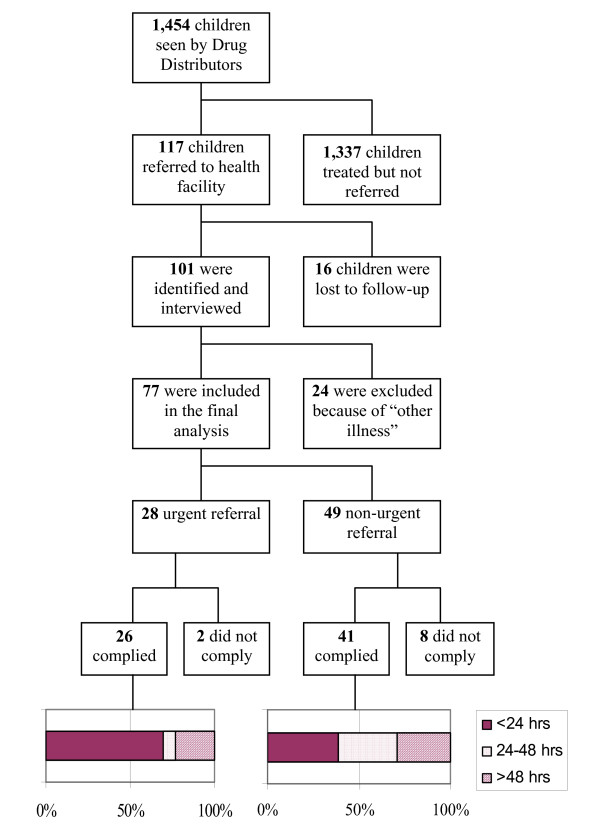
Schematic presentation of study sample, including types of referral, referral completion and timing of referral for 1,454 children under-five seen by Drug Distributors.

During the 20 study weeks 1,454 children sought care from the DDs. Sixty-eight percent (994/1,454) sought care within 24 hrs of fever onset. The overall referral rate was 8% (117/1,454), varying between 3% and 12% across parishes. Among those referred 16 (14%) were lost to follow up resulting from hospital admission >30 days, migration, or ending of study. Children lost to follow-up did not differ in age or sex. Of the 101 children identified and caretaker interviewed, 24 were excluded because of 'other illnesses'. Of the remaining 77 referred children included in the final analysis, 36% (28/77) were referred urgently and 64% (49/77) non-urgently (Figure 1). The median age of children seen by DDs was 24 months (range 0–96 months) but was lower among those referred (18 months) than those not referred (24 months) (Wilcoxon rank-sum, p = 0.006).

Mothers mainly sought care for their child because of fever (93%; 94/101), general illness symptoms (85%; 86/101) and various ARI symptoms (30%–66%), Table 1. DDs mainly referred persisting fevers (32%; 32/101), convulsions (15%; 15/101) and vomiting (11%; 11/101). Thirty-two percent (24/76) of caretakers complained of fast breathing in the child. Of these, 50% (16/32) were 'urgently referred'. Thirty percent (30/101) complained of 'Ekyikenyera' – a local illness concept including rapid breathing, chest indrawing and high fever – but only 4 DD referrals were due to this illness.

**Table 1:** Mothers' reported symptoms prompting care seeking and referral reasons recorded by Drug Distributors

Symptom	**Mothers' reporting (n = 101)**	**DD's reporting (n = 101)**
	
	n (%)*	n (%)*
Fever	94 (93%)	-
No recovery/persisting fever	-	32 (32%)
General illness symptoms	86 (85%)	2 (2%)
Cough	67 (66%)	9 (9%)
Not able to eat/breast feed	55 (54%)	4 (4%)
Vomiting	36 (36%)	11 (11%)
Fast breathing	24 (32%)†	-
'Ekyikenyera'^‡^	30 (30%)	4 (4%)
Diarrhoea	25 (25%)	6 (6%)
Convulsion	14 (14%)	15 (15%)
Over/under age	-	5 (5%)
Unable to sit or stand	-	2 (2%)
Loss of consciousness	-	1 (1%)
Other conditions^#^	16 (16%)	24 (24%)

*Many children had more than one symptom and received more than one diagnosis by the DD, therefore percentages add up to more than 100%

†The local terms for fast breathing and difficult breathing were added to the questionnaire at a later stage; hence the percentages are based on a smaller denominator (n = 76)

^‡^The local illness 'Ekyikenyera' includes symptoms of grunting, stridor and chest indrawing^3^

^#^Chickenpox (7), conjunctivitis (4), wounds/burns (3), undefined illness (4)

Overall, 87% (67/77) of referred children completed referral by being taken to either a hospital or health centre (Figure 1). Referral completion rate ranged from 84% (11/13) to 100% (6/6) across parishes and was 93% (26/28) for 'urgent referrals' versus 84% (41/49) for 'non-urgent referrals' (p = 0.31). Lack of money (5/10) and child improvement (4/10) were main reasons for non-completion. Instead, 5 were taken to a drug shop and the rest did nothing. At the time of the interview, 3 of the 10 non-compliers and 13 of 67 compliers had still not recovered. 'Urgent referrals' were more likely to access referral care within 24 hours (69%; 18/26), compared to 'non-urgent referrals' (39%; 16/41) (p = 0.016). Children <1 year were more frequently taken for timely referral than children ≥ 1 year (p = 0.25). Lack of money and waiting for malaria drugs provided under HBM to finish were main reasons for delay.

Of those stating referral completion 82% (55/67) were identified in health facility registers: 84% (46/55) were diagnosed with malaria; 42% (23/55) were diagnosed with ARI, whereof 78% (18/23) were prescribed an antibiotic. Only some health workers separated upper ARI from lower ARI (LRI). Nevertheless, 26% (6/23) of ARIs were diagnosed as LRI. More than half of the ARIs (13/23) and 4 out of 6 LRIs had waited ≥ 2 days before being seen in a health facility.

Most referred children went to governmental health centres (37%; 25/67) or hospitals (37%; 25/67) and 26% (17/67) were taken to non-governmental (NGO) or private health centres. Thirty-eight (10/26) of 'urgent referrals' were taken to NGO/private health centres compared to 17% (7/41) of 'non-urgent referrals' (p = 0.05). Median (range) out-of-pocket cost of referral was US$ 10.00 (4.40–64.70) for the NGO hospital, 1.76 (0.47–19.40) for NGO/private health centre and 0.71 (0.00–5.00) for governmental health centres. Overall, 60% of the total cost went to consultation/admission fees. The money was obtained with difficulty for 71% (45/65) of the caretakers and 62% (40/65) had sold household assets to finance the referral event.

## Discussion

We demonstrate that 87% of children referred in the Ugandan Home Based Management of fever strategy (HBM) reach a health facility. The high community referral compliance rate observed indicate that it is possible to extend the health system to the village to catch likely malaria episodes early while still having potentially severe cases reaching the health facility for assessment [19].

Although this isa different kind of referral (community to health centre) the main barriers to successful referral, i.e. lack of money and mother not being told to go for referral immediately, are the same as those outlined in other papers for health centre to hospital referral [16,20,21]. Referral compliance could potentially have been improved by the use of referral slips or "counter-referral" slips, with feedback to the DD from the health facility [6,20]. In addition, lack of regular DD supervision may have contributed to unsuccessful referral. Community financing schemes or other mechanisms to improve financial access to referral care have been proposed as life-saving strategies to facilitate emergency care seeking in remote and resource-poor health infrastructures [22,23]. While these risk-sharing strategies primarily have focussed on emergency transport [22], such financing schemes would also need to address issues of access to cash for expenses such as buying drugs and food, since an important source of delay in accessing referral care is having to raise cash by selling crops or belongings before going [16].

Our findings of high compliance rates are similar to reports on referral from community to health centre in Mali [23] but contrasts with the low 28% referral completion rate from Ugandan health centres to hospital [16]. This is a plausible pattern since seeking care from the health center may already have exhausted the family's resources, making a further referral step to hospital wholly unrealistic, primarily for lack of money [16]. The low compliance rate in this latter stage of referral to hospital therefore is an argument for e.g. Home Management strategies which would catch children early and prevent development of severe disease. While community based studies need to assess the degree to which children are treated earlier under HBM, we are still encouraged to find that 68% of children in the Drug Distributors' records were stated to have been sick for less than 24 hours.

However, compliance with referral advice is still not prompt: almost 1/3 of 'urgent referrals' delayed ≥ 24 hours before accessing referral care, in line with findings from heath centre referral in Tanzania [17]. This is worrisome since timely referral often is key to preventing mortality in severely ill children [24]. The main reason for delay was waiting for the 3-day course of antimalarials to finish – likely explaining why the majority of ARI cases seen in referral health facilities came 2 days after visiting the DD. The antipyretic effect of Chloroquine on febrile illnesses other than malaria could potentially have contributed to delaying referral care-seeking. Whether such "maltreatment" of ARI with antimalarials affects the disease progression to severe ARI and treatment outcome needs to be explored in clinical studies. However, delayed care seeking and inadequate referral for ARI have been shown to be important predictors for child deaths in Mexico [25].

We found that the Drug Distributors had recorded and referred few children with complaints of lower ARI symptoms. This could be explained both by lack of mothers' reporting [26] and by DD failure to identify the symptoms [27]. The clinical resemblance of malaria and mild pneumonia may also explain why such cases receive Homapaks without referral advice [28]. Furthermore, it was observed that in the local HBM guidelines, the referral symptom "difficult breathing" had been translated into 'Ekyikenyera'- a local illness classification which likely correlates with severe pneumonia [13]. This may be too restrictive and not include referral of children with increased respiratory rate, a sign of less severe pneumonia. Hence, mothers' and DDs interpretation of the local term for 'fast breathing' needs to be explored as well as their ability to identify early pneumonia symptoms and make presumptive diagnosis based on respiratory rate.

The finding that children <1 year were more likely to access timely referral care, contradicts previous findings of bias in allocation of household resources against young children [29], and deserves further exploration.

Community and Drug Distributor knowledge of the study could have influenced attendance, referral and compliance rate, and our results may reflect "better-than-average" performance, not generalisable to the whole country. Assessing behaviour based on mothers' *stated *practices also limited the study, but the information collected from health facility records partly validated their reporting. We also had no means of verifying the quality of the Drug Distributors' recorded information. However, the study was conducted under difficult circumstances and despite methodological difficulties, a relatively small study sample, and the potential bias introduced, this study is one of the very few that investigate this new type of referral. This study design has also been judged most appropriate for the study purpose [24] and merits to be repeated in other areas implementing home management strategies. Given the lack of diagnostic equipment (microscopy and x-rays) in most rural health facilities, health worker diagnosis was presumptive, thus the clinical value of diagnosis cannot be ascertained.

## Conclusion

In conclusion, this study confirms that the Ugandan HBM strategy can achieve high community referral compliance and thus maintains linkage with formal health services. Although failure to recognise symptoms of possible pneumonia and delays in referral care access for respiratory illness symptoms may pose hazards for children with acute respiratory infections, referral non-completion was not a major issue in this specific setting. Our findings imply that the WHO/UNICEF recommendation to include pneumonia in community management of malaria [30] may reduce the risk of mismanagement and increase prompt and effective care of the sick child in sub-Saharan Africa.

## List of abbreviations

HBM – Home Based Management of fever

DD – Drug Distributor

ARI – Acute Respiratory Infection

LRI – Lower respiratory Infection

## Competing interests

The author(s) declare that they have no competing interests'

## Authors' contributions

KK participated in the conception, design, acquisition, analysis and interpretation of data, drafting the article and revising it critically. GT participated in the conception, design, interpretation of data, drafting the article and revising it critically. JNS participated in the design, interpretation of data, drafting the article and revising it critically. YS participated in the design, acquisition of data and revising the article critically. GP participated in the design, interpretation of data, drafting the article and revising it critically. SP participated in the conception, design, analysis and interpretation of data, drafting the article and revising it critically.

## Pre-publication history

The pre-publication history for this paper can be accessed here:


